# Young People’s Use of Digital Health Technologies in the Global North: Narrative Review

**DOI:** 10.2196/18286

**Published:** 2021-01-11

**Authors:** Deborah Lupton

**Affiliations:** 1 Vitalities Lab Centre for Social Research in Health and Social Policy Research Centre UNSW Sydney Kensington Australia

**Keywords:** digital health, young people, Global North, social research, narrative review

## Abstract

**Background:**

A diverse array of digital technologies are available to children and young people living in the Global North to monitor, manage, and promote their health and well-being.

**Objective:**

This article provides a narrative literature review of the growing number of social research studies published over the past decade that investigate the types of digital technologies used by children and young people in the Global North, in addition to investigating which of these technologies they find most useful or not useful. Key findings as well as major gaps and directions for future research are identified and discussed.

**Methods:**

A comprehensive search of relevant publications listed in Google Scholar was conducted, supported by following citation trails of these publications. The findings are listed under type of digital technology used for health: cross-media, internet, social media, apps and wearable devices, sexual health support and information, and mental health support and information.

**Results:**

Many young people in the Global North are active users of digital health technologies. However, it is notable that they still rely on older technologies, such as websites and search engines, to find information. Apps and platforms that may not have been specifically developed for young people as digital health resources often better suit their needs. Young people appreciate the ready availability of information online, the opportunities to learn more about their bodies and health states, and the opportunities to learn how to improve their health and physical fitness. They enjoy being able to connect with peers, and they find emotional support and relief from distress by using social media platforms, YouTube, and online forums. Young people can find the vast reams of information available to them difficult to navigate. They often look to trusted adults to help them make sense of the information they find online and to provide alternative sources of information and support. Face-to-face interactions with these trusted providers remain important to young people. Risks and harms that young people report from digital health use include becoming overly obsessed with their bodies’ shape and size when using self-tracking technologies and comparing their bodies with the social media influencers they follow.

**Conclusions:**

Further details on how young people are using social media platforms and YouTube as health support resources and for peer-to-peer sharing of information, including attention paid to the content of these resources and the role played by young social media influencers and microcelebrities, would contribute important insights to this body of literature. The role played by visual media, such as GIFs (Graphics Interchange Format) and memes, and social media platforms that have recently become very popular with young people (eg, Snapchat and TikTok) in health-related content creation and sharing requires more attention by social researchers seeking to better understand young people’s use of digital devices and software for health and fitness.

## Introduction

Over the past decade, an expanding array of digital technologies have emerged that can be used for promoting or managing young people’s health and physical fitness. These devices and media are often referred to as *digital health* technologies. In addition to established digital media, such as online discussion forums and websites about health topics, telemedicine, and exergames, these technologies include new media and devices, such as social media for the discussion of health topics, apps for mobile devices, and wearable devices, including smartwatches or fitness bands that assist with monitoring information about bodily activities and functions [1]. Children and young people are now often encouraged by their parents or teachers to use these devices and software designed to promote their health and fitness [2-5].

In response to these newer digital health technologies, a growing body of social research has developed to investigate how young people living in countries that are often characterized as *the Global North* are engaging with them. The Global North is a term used in the social sciences and humanities to refer to politically stable and wealthy countries or regions that have a western cultural orientation (ie, Western Europe, North America, the United Kingdom, Australia, and New Zealand). As is the case with research, in general, on young people’s use of digital technologies [6], far more research has been published—at least in the English language—on their engagement with health-related media and devices in the Global North compared with other regions.

This article presents a narrative review and synthesis of the most recent literature, published from 2010, on young people’s use of digital health technologies in the Global North. It includes relevant studies in disciplines such as sociology and media studies that are not often considered in medical or public health research.

The research questions structuring the narrative review were as follows:

1. What digital media and devices are used for health-related purposes by children and young people, aged 5 to 30 years, living in countries in the Global North?
2. Which of these media and devices do they find most useful and helpful?
3. What are the age-related, gendered, socioeconomic, cultural, and geographical dimensions structuring their use or nonuse of digital health?
4. What risks and harms are associated with young people’s digital health use?
5. What are the gaps in the social research literature on this topic?

## Methods

There are various approaches to narrative literature reviews. The approach adopted here is not the hybrid *systematic narrative review* that is beginning to appear in the body of reviews focusing on medical internet studies. Instead, this narrative review draws from the long-established tradition of literature reviews in social inquiry. The purpose of this type of narrative review is to provide a scholarly summary and interpretation of a body of literature rather than evaluate the validity of a method or a medical intervention, with the intention of integrating and deepening understanding of a particular research topic [7]. The aim is to provide a synthesis of what is known about the chosen topic and identify research gaps [8]. It is particularly useful for synthesizing the scholarly literature in the humanities and social sciences—the disciplines encompassed in this review.

This narrative review method does not attempt to adopt a quantitative approach focused on *rigor*, *avoidance of bias*, and *interrater reliability*, rather it focuses on a qualitative interpretation and synthesis of the state of knowledge in a selected body of literature on a defined topic [7]. It is a different and complementary approach to the systematic review and is an appropriate approach to adopt for a review that is directed at social research [7].

The approach taken for this narrative review is similar, for example, to that undertaken for an assessment of the literature on the role played by gangs in adolescent mental health [9] and the health effects of video gaming [10]. Iterative searches of Google Scholar were conducted to find relevant publications published in English, using the search terms “children,” “young people,” “teenagers,” and “adolescents” in combination with “digital health” and then, more specifically, combined with “health,” “digital media,” “websites,” “telemedicine,” “telehealth,” “electronic patient records,” “social media,” “apps,” “smartphones,” “wearable devices,” “exergames,” “Facebook,” “YouTube,” “Instagram,” “Pinterest,” “Twitter,” and “Snapchat.” Google Scholar was chosen as the database to search because, unlike other major databases such as Web of Science, PubMed, or Scopus, it is far more inclusive of humanities and social sciences outputs, such as books, book chapters, and journals, which are not included in science- or medical-based databases, while simultaneously including the publications listed in those databases [11].

I read all of the publications found using these searches, including books, book chapters, reports, and journal articles. I then followed the citation trails, identifying and reading outputs that cited the initial identified studies. Only publications appearing since 2010 were included, as I wanted to limit the review to research that considers the most recent digital technologies for health. As an initial search demonstrated, there has been quite a lag in research on young people’s use of digital health, with most relevant studies published since 2015. Publications were not included in the review if they did not have a predominantly social research focus or if they did not specifically focus on children or young people’s use of digital technologies for health-related purposes (eg, publications about digital health use by parents or health care providers on behalf of young people, or those concerning evaluations of the accuracy of digital health software or devices). Only those publications referring to children or young people living in the Global North were included. Using these criteria, a total of 71 publications were identified for inclusion in this review.

Discussion of the literature review is organized by type of digital technology, beginning with studies presenting a broad overview of digital technology use for health across devices and software, followed by more specific topics: use of cross-media, internet, social media, health apps, and wearable devices. There is also discussion of the literature on sexual health support and information as well as mental health support and information, as these health areas were prominent in the social research on young people’s use of digital health. The Discussion section summarizes the key insights and major gaps identified in the literature and suggests directions for future social research studies.

## Results

### Cross-Media

Only a small number of studies have investigated young people’s use of digital technologies for health-related purposes across the range of diverse media and devices currently available to them. One of the earliest is a national representative survey of young people, aged 13 to 18 years, that was conducted in 2014 in the United States [12]. Findings highlighted the continuing importance to young people of face-to-face encounters with family members and health professionals. While the respondents did report searching online for health information, they turned to their parents more often, as well as to doctors and nurses. Furthermore, most respondents reported that they did not frequently use the internet for health information. When they did use the internet for this purpose, they tended to research fitness, diet, or nutrition topics. This survey found that older, wealthier, more active, and healthier young people searched more frequently, as did African American and Hispanic adolescents. It is notable that most respondents did not use social media when searching for health information. A total of 1 in 5 respondents had used YouTube for health topics, which was more than those who used Facebook or Twitter. Among the respondents possessing a smartphone, 29% had downloaded a health app, with fitness or exercise apps being the most common. However, almost half of those who had downloaded a health app hardly ever or never used it. Most respondents had never used a wearable device for health-related purposes.

Another more recent US-based project using qualitative methods found that young people aged 13 to 18 years living in Seattle reported three main uses of digital technology for health: (1) to gather information, such as from medical websites, social media (ie, Facebook and Pinterest), exercise apps, and YouTube for workout information; (2) to share experiences and view others’ experiences to gain social support or inspiration; and (3) to engage in self-tracking for health (ie, apps and wearables) [13]. This research found that social media was not a preferred mode to communicate with health care providers for the participants because of privacy and intrusiveness concerns. Online media and apps were considered easy to use, always available, nonjudgmental, and to have options to share information and experiences anonymously. Negative aspects identified by these young people included online health information not always being credible, not being able to afford some health apps and wearable devices, finding it burdensome to enter information into apps, being tempted to always have the phone on and being distracted, technology not being designed for teenagers’ use, and peers sharing health information that may be inaccurate or may encourage risk-taking.

Social research in other English-speaking countries in the Global North has indicated high use of the internet, apps, and wearable devices for health-related purposes among young people but also continuing support of long-standing digital platforms and tools. A 2017 study interviewing young Australians, aged 16 to 25 years, found that they valued the convenience, accessibility, detail, and diversity of health information offered by digital media and devices [14]. These young people were active users of older technologies for health, such as search engines and websites, even more than of new technologies, such as social media, apps, and wearable devices. Similar to the respondents in the US survey by Wartella and colleagues [12], these young Australians continued to value personal connections with other people for providing health information and support, including family members and friends as well as medical professionals.

A synthesis of findings across three English-based projects involving both qualitative and quantitative investigations among secondary school students reported on the findings relating to the use of social media, apps, and wearables for health-related purposes [15]. These studies demonstrated that these young people were both critical and vulnerable users of these devices and software. The participants reported a range of positive benefits, particularly in relation to supporting promotion of healthy diet, exercise, and positive body image. However, they also raised concerns about the power of these technologies to shape, influence, and change their health-related behaviors.

Research among secondary school students in South West England in a 2017-2018 study, using a survey as well as qualitative methods, found that the participants were actively using a range of digital devices and software for health-related purposes [16]. Survey data showed that smartphones were the devices most often used to access health information, and half of the respondents were using mobile apps to track their health, diet, or fitness levels. YouTube was the most popular source of health information: 44% of survey respondents reported use of this platform. Fitness was the most popular category of health-related videos watched by participants. Many participants also reported following content by social media influencers about fitness and diet regimens, often organized around the “fitspiration” or “fitspo” hashtags. However, these young people also reported that they considered official websites, such as those offered by government agencies, as the most helpful source for learning about health.

Despite these high levels of digital health use, the participants in these Australian and English studies often noted that it could be difficult to access the accuracy of health information online among the multiple and varied sources available to them and sought help from trusted adults to negotiate this information. However, they demonstrated little notable interest or concern about commercial third parties who might be accessing or exploiting their health data. Participants in the English study did express caution about the people in their lives with whom they knowingly shared their personal health information. Most were willing to share their health data with their parents but to a much lesser extent with health professionals or their friends. 

### The Internet

Search engines, websites, and online discussion forums were among the earliest forms of online tools for finding and sharing health information. As outlined in the above section, recent research suggests that these resources remain popular for young people in the Global North. Other studies also support these findings. A systematic review of 19, mostly US-based, studies on young people’s health-related internet use identified evidence of adolescents seeking preventive health care information, support, and specific information about common health issues, such as sports injuries, flu, chronic diseases, asthma, sexual health, fitness, and infections [17]. The internet served as a confidential source of information that was particularly appreciated by youth facing limited access to health care or managing cultural or religious sensitivities. The value for young people seeking help and support from peers online for mental health problems, living with chronic illnesses, and connecting with young people with the same sexual orientation was a common finding across these studies. Gender and age structured young people’s digital health practices. Girls tended to use the internet more often than boys. Older youth preferred online sources of health information, while younger people sought help primarily from their parents, teachers, and other adults. Overall, the young people in these studies perceived their online experiences as positive, but they expressed concern about online privacy and the accuracy of the information they were accessing.

Another systematic review assessed studies of how adolescents search for and evaluate health information online [18]. Four themes were identified across the literature: (1) use of search engines, (2) difficulties in selecting appropriate search strings, (3) barriers to searching, and (4) absence of searching. The review concluded that adolescents are aware of the varying quality of online information about health and exhibit a range of strategies for searching and appraising this information. However, one of the most significant barriers to successfully finding information is the volume of search returns they often encounter, sometimes resulting in them giving up in frustration.

A French survey found that almost half of the young internet users, aged 15 to 30 years, in France searched online for health information. Female participants and those of higher socioeconomic status were more likely to do so [19]. In another more recent French survey involving students aged 18 to 24 years, almost all of the students had searched on the internet for health-related information in the past 12 months, suggesting a growing reliance on the internet among this demographic group in France [20].

### Social Media

In the past 5 years, a growing number of social researchers have investigated how young people in the Global North use social media for health-related purposes. Analyses of the content of youth-oriented social media [20-22] and visual media, such as selfies [23,24] and the use of hashtags [25,26] as a way of organizing communities of young people around topics including fitness, sporting activities, health, diet, sexuality, gender identity, and self-care, have received attention in the literature. Researchers have demonstrated that social media influencers, microcelebrities, and other content creators, many of whom are young people themselves, are playing important roles in conveying health information and providing emotional support to young people [14-16,26-29].

The “fitspiration” or “fitspo” hashtags used with images intended to show how physical exercise and training can sculpt bodies, particularly on Instagram, have received particular attention, with researchers showing how young women’s bodies are depicted as sexualized, normatively slim, toned, and typically White, while young men are shown as conforming to the muscular or hyper-muscular ideal [30-34]. The relationship between the consumption of digital media and young people’s body image has also been explored in several studies, with some evidence to suggest that such images can influence young people’s appraisal of, and satisfaction with, their body size, shape, and level of fitness [16,27,35-39]. Researchers have also raised concerns about the promotion of health-detracting practices in social media and YouTube videos, such as self-harm [38] and disordered or overly restricted eating [31,33,39-43].

Goodyear and colleagues used participatory class activities, interviews, focus group discussions, and an online survey with English students, aged 11 to 15 years, to explore their engagement with health-related social media across the range of platforms available to them [44]. Just over half of the survey respondents actively used social media to search for health information, predominantly for physical activity, diet and nutrition, and body image. Almost half (46%) of the respondents said that they had changed their health-related behaviors because of something they had seen on social media; most of them said the change was positive. There were five types of content these participants identified as influential: (1) automatically sourced (ie, pushed to them by the platform’s algorithms, including advertising material), (2) suggested or recommended following an initial search, (3) peer generated, (4) likes (ie, positive responses from others regarding their content, suggesting it was good), and (5) reputable content (ie, from official organizations, celebrities, sports figures, big name commercial companies, etc).

A study using focus groups and a survey included adolescent girls living in the US state of West Virginia and queried them about their use of health-related social media [45]. The findings demonstrated the popularity of Snapchat and Instagram with this age group as social media platforms to communicate with friends, with Facebook proving unpopular and viewed as “for older people.” In terms of health-related content, these platforms were used to share nutrition information, healthy recipes, weight loss, and fitness posts with friends.

### Apps and Wearable Devices

Several systematic reviews have been published of studies investigating various aspects of young people’s use of health apps. One focused on studies of adolescents’ use of apps for chronic disease management, finding that there was a paucity of evidence-based apps and very few studies evaluating their effectiveness [46]. A systematic review of research on apps used for health promotion among adolescents and students found that most apps were used for therapy or as part of school programs, and only four of them had been specifically designed for adolescents. The review identified that limited research has been conducted on how effective these apps are for promoting health among young people [47].

In-depth research has been able to bring to the surface some of the reasons why young people choose to use health or fitness apps and what aspects of the apps they find most beneficial or helpful. Interviews with American college students who used health and fitness apps found that most of the students had downloaded the app to help with meeting goals, which included supporting an established behavior or adopting a new behavior. They liked apps that were free, easy to use, provided visual and auditory cues, and had game-like rewards, but they did not want to link the apps to social media [48]. Findings from an English study that involved a survey, workshops, and interviews with young people aged 18 to 25 years showed that a calorie-counting app was the most popular and was used by almost half of the participants [49]. Many participants had discontinued using a health or fitness app, however, citing lack of interest, time, or motivation as reasons. Some expressed concern that they were becoming obsessive about counting calories or overexercising, or they struggled with feelings of guilt, anxiety, failure, or disappointment in response to the metrics generated by the app. It was noted by some participants that they felt a need to “reconnect” to their bodies without using digitized and quantified data to generate an understanding of their fitness and health. Similar findings were reported in an interview-based study with young New Zealanders aged 16 to 21 years [50].

Another English-based project involving focus group discussions about fitness apps with adolescent girls who were sports leaders at their school also demonstrated feelings of ambivalence on the part of the girls [51]. These students were mostly positive about using such apps for motivating young people to improve their fitness and health. However, they cautioned that physical education classes should not rely on these kinds of technologies and that the social benefits of exercising with others could be lost.

Far less research has directed attention to young people’s use of wearable devices for health and fitness. Studies that have been published suggest that health and fitness apps on smartphones remain more popular than wearable devices among young people. A systematic review of the feasibility and effectiveness of wearable activity trackers among children and young people found that few studies had been conducted. This research suggested that while intervention effects from wearable device use were generally positive, they largely related to short-term rather than long-term effects. Feasibility studies indicated that comfort, design, and feedback features were important [52].

Gender differences were identified in a French survey of students aged 18 to 24 years. The survey found that 35% of the students had used at least one health app, mostly for physical activity and general health monitoring, but only 4% had a wearable device. Female students were more likely to use a health app, while male students were more likely to use a wearable device [20]. In a Belgian survey of adolescent high school students, researchers found that one-quarter of them used nutrition or fitness apps or both. Those who used these types of apps more frequently were more likely to consume healthy beverages and have a lower BMI; however, those who used nutrition apps the most were more likely to have a higher BMI, probably because they were trying to lose weight [53].

Two studies investigated Finnish young people’s use of apps and wearable devices for fitness-related purposes. One survey found that half of the respondents said they had these types of apps on their phones, but only 16% reported using them. Only 17% owned wearables and few participants (9%) used them [54]. In the second project, a more specific group of Finnish young people (ie, senior high school athletes) reported a high voluntary use of self-tracking apps and wearable devices to monitor their training and fitness levels, to the point that the use of such technologies was positioned as an important dimension of their identities as accomplished athletes [55].

A small number of projects involved providing young people with fitness trackers to ascertain how they used them. In one such study, Australian adolescents, aged 13 to 14 years, were given a Fitbit Flex wristband and app and were asked to use it for 6 weeks [56]. The participants reported finding it easy for tracking physical activity but not as easy to use for sleep. Barriers to use included its lack of comfort and design, lack of specific feedback about activity levels, and inability to use it in water-based sporting activities. Another Australian study involved providing younger children, aged 7 to 12 years, with an activity tracking device (ie, KidFit) for a period of 4 weeks and included interviews with their parents [57]. Children and parents reported that they found the associated app easy to use; however, the children were frustrated by not being able to receive real-time feedback, and there were difficulties for some with the band feeling uncomfortable and having to remove it for water-based sports activities.

In a US-based research study, young people enrolled in an after-school program were provided with Fitbits for a period of 6 months [58]. Data logs revealed low continuous engagement that declined over time. When the young people did wear their Fitbits, they engaged with their data in reflective ways. The design of the Fitbit, environmental constraints, and motivation were barriers to continuous engagement.

Two projects invited English young people to try Fitbits. One study involved 100 young people aged 13 and 14 years [59,60]. The researchers noted that the participants’ motivation to use the wearable devices faded quite quickly, as they became bored with them and disliked the surveillance opportunities the devices afforded their teachers. The young people felt pressured by the demands of the step-counting goals set by the devices. They questioned the value of the metrics generated by their Fitbits and resisted the ways the devices tended to limit physical activity and health status to certain defined measurements, such as step counts.

The second English study adopted a combination of Fitbits and the WhatsApp messenger app to investigate young people’s experiences with the devices [16]. A total of 2 girls and 5 boys, aged 16 to 18 years, used the Fitbits for 8 weeks. Several of the participants enjoyed counting their steps, reviewing their metrics instantaneously, competing with others, reaching goals and targets, and receiving positive notification from the devices. For some, the devices generated motivation and short-term behavior changes. Others struggled with knowing how best to respond to the data generated by their Fitbits without becoming too obsessive about their metrics and goals, such as loss of body weight; some simply found the process of remembering to use the devices and to input data or understanding what the metrics were telling them as involving too much labor on their part. As in the other English study, the participants recounted losing the motivation to wear their Fitbits or becoming annoyed with being “nagged” by them to be more physically active.

### Sexual Health Support and Information

Health promotion agencies often seek to develop digital resources to support young people’s sexual health. However, these efforts tend to ignore the multiple ways in which a broader ecosystem of digital media and devices are operating in ways that are preferred by young people [61]. These include practices such as consensual sexting, peer-to-peer sharing of information in websites, social media and online discussion forums, and YouTube videos as well as news coverage of sexual health issues on alternative youth-oriented news platforms, such as Buzzfeed, Broadly, and Vice [61-63]. Searching online tends to be the first port of call for young people when they have sexual health concerns [64] or simply want more information about sex [65-67].

Research with LGBTQI (lesbian, gay, bisexual, transgender, queer, and intersex) youth has found that they consider apps that have been specifically designed to support their health and well-being to be “pretty pointless” [68]. In contrast, personal stories are valued by young people looking for information about sexual identity [65]. In particular, the social media platform Tumblr has been an important resource for LGBTQI youth to find connections with others and learn about their sexual identities [69-71]. This opportunity has recently been closed down, however, with Tumblr introducing a policy that it would no longer host “adult content,” thereby effectively censoring much LGBTQI content [69].

### Mental Health Support and Information

Many websites, online programs, and apps have been created specifically to support young people’s mental health. A systematic review of studies devoted to the use of mental health apps for children and adolescents showed that very little research has been conducted into their effectiveness [72]. The few studies that have been published failed to demonstrate a significant effect on intended outcomes. Another systematic review focused on research that investigated young people’s online health-seeking behaviors for mental health concerns [73]. Across the included studies, key benefits included anonymity and privacy, immediacy, ease of access, inclusivity, the ability to connect with others and share experiences, and a greater sense of control over the help-seeking journey. Online help-seeking has the potential to meet the needs of those with a preference for self-reliance or act as a gateway to further help-seeking. Barriers to help-seeking included a lack of mental health literacy, concerns about privacy and confidentiality, and uncertainty about the trustworthiness of online resources.

Young people’s mental health and well-being may be supported through digital media that have not been specifically designed for this purpose, and young people themselves often play active roles in generating, curating, and sharing content for these services. For instance, platforms like YouTube, Facebook, Twitter, Tumblr, and Instagram host discussions of topics related to health and well-being, such as mindfulness, stress relief, healthy eating, and physical fitness, as well as provide opportunities for young people to create informal networks to share their experiences of specific mental health conditions and provide information and emotional support to each other [74]. Social research studies have shown that informal online communities developed by young people with peers can promote recovery from conditions such as eating disorders [75], depression [76], suicidal ideation, and self-harm [77].

Online discussion forums have been demonstrated to provide help and support to many young people with mental health conditions or difficulties [78]. A study using focus groups with Australian university students reported that the students appreciated internet-based resources of mental health information and support, particularly for their accessibility and the anonymity and avoidance of stigma these offered [79]. However, they were also concerned about sensitive details about their mental health being accessed by third parties and found the wealth of information distributed across the internet to be difficult to locate and assess for its relevance. A desire for a centralized resource was expressed by these participants.

A Canadian online survey of young people aged 17 to 24 years [80] found that most respondents had used the internet to seek information or help for distressing feelings. Information-based websites were popular, followed by social media sites. Privacy was rated as very important by these Canadian respondents, and they particularly valued information about interventions and treatments. New Zealand–based interviews with a group of participants aged 16 to 21 years noted the importance of mindfulness and meditation apps and trusting relationships with peers on social media for providing relief from distress [50,81].

## Discussion

Returning to the research questions addressed by this review, the following trends and gaps were identified across the body of reviewed literature.

### What Digital Media and Devices Are Used for Health-Related Purposes by Children and Young People, Aged 5 to 30 Years, Living in Countries in the Global North?

The reviewed research has found that many young people in the Global North are active users of digital health technologies. However, it is notable that they still rely on older technologies, such as websites and search engines, to find information. In many cases, newer media, such as social media platforms, apps, and wearable devices, are less frequently used by young people for health-related purposes. Several studies have demonstrated that young people who have experimented with the use of self-tracking apps and wearable devices do not necessarily continue using them on a long-term basis.

### Which of These Media and Devices do They Find Most Useful and Helpful?

Young people in this body of research have reported many benefits from their use of digital technologies for health. They appreciate the ready availability of information on the internet and the opportunities to learn more about their bodies and health states and how to improve their health and physical fitness by accessing websites, discussion forums, and social media and by using apps.

An important finding across the literature is that apps and platforms that may not have been specifically developed for young people as digital health resources (eg, YouTube, Tumblr, and Instagram) often better suit their needs. Young people appreciate being able to connect with peers and find emotional support and relief from distress by using social media platforms, YouTube, and online forums. They can find self-tracking apps and platforms helpful for learning more about their bodies. However, they can become bored, feel overwhelmed by the vast quantities of data generated by these technologies, or find the labor involved in self-tracking as too burdensome for continuing use.

Another key finding across some of the reviewed research is that young people can find the vast reams of information available to them on the internet to be difficult to navigate. They often look to trusted adults, particularly their parents and medical professionals, to help them make sense of the information they find online and to provide alternative sources of information and support. Face-to-face interactions with these trusted providers remain important to young people.

### What Are the Aged-Related, Gendered, Socioeconomic, Cultural, and Geographical Dimensions Structuring Young People’s Use or Nonuse of Digital Health?

Published studies have mostly been based in the United States and the United Kingdom, but there are also some publications from researchers in Western Europe, Australia, New Zealand, and Canada. Little research thus far has focused on comparing the experiences of young people based on sociodemographic attributes, such as their age, geographical location within a country (eg, in rural or remote regions compared with urban regions), gender, socioeconomic status, and race or ethnicity. Those studies that have made these comparisons have identified that these factors can play an important role in structuring the ways that young people engage with digital health. It is notable that the experiences of preadolescent children have received little attention; most studies published thus far have focused on teenagers and young people aged in their early 20s.

### What Risks and Harms Are Associated With Young People’s Digital Health Use?

Risks and harms that young people report from digital health use include becoming overly obsessed with their bodies’ shape and size using self-tracking technologies and comparing their bodies with the social media influencers they follow. The implications of personal data exploitation for children’s rights [82-84] and citizenship [85] have also been noted by media researchers. Major organizations directed at supporting children’s rights and well-being, globally, are beginning to draw attention to the opportunities and risks involved in introducing digital technologies into children’s lives [86]. Young people often have little choice in engaging with datafication technologies [87]; for example, when they were expected to use digital learning platforms, biometric systems, or self-tracking devices at school, there was little or no option to opt out [2,3,82]. These technologies can limit young people’s privacy, agency, and autonomy [88-91].

### What Are the Gaps in the Social Research Literature on This Topic?

More comparative research needs to be conducted on how young people from different sociodemographic backgrounds and age groups experience digital health. There is a wide scope for further research that can address these differentiated groups of young people. Only a small number of studies have directly asked young people about their practices or attitudes concerning their health data privacy and security. This research has found that many young people are not overly concerned about these issues, except where it relates to highly sensitive topics, such as sexual or mental health. Young people tend to lack knowledge about the third parties who access their personal health and medical data and what these actors and agencies do with their health information. Given the high prevalence of personal health data hacks, breaches, and leaks [92-94], as well as third-party use of this information across age groups [95-98], what knowledge this age group has about these issues and how they can better learn about and become aware of what happens to their health data require further research attention.

Young people’s use of social media and apps has received reasonably high levels of attention by social researchers. However, further details on how young people are using social media platforms and YouTube as health support resources and for peer-to-peer sharing of information, including attention paid to the content of these resources and the role played by young social media influencers and microcelebrities, would contribute important insights to this body of literature.

Finally, the role played by visual media, such as GIFs (Graphics Interchange Format) and memes, and social media platforms that have recently become very popular with young people (eg, Snapchat and TikTok) in health-related content creation and sharing requires more attention by social researchers seeking to better understand young people’s use of digital devices and software for health and fitness.

### Limitations

This review was limited to research that adopted a social perspective, was published in English, and was conducted with young people living in countries in the Global North. Future reviews could address other bodies of research beyond these parameters to supplement the findings of this review.

## Abbreviations

GIF: Graphics Interchange Format
LGBTQI: lesbian, gay, bisexual, transgender, queer, and intersex
